# PID Control as a Process of Active Inference with Linear Generative Models †

**DOI:** 10.3390/e21030257

**Published:** 2019-03-07

**Authors:** Manuel Baltieri, Christopher L. Buckley

**Affiliations:** EASY Group—Sussex Neuroscience, Department of Informatics, University of Sussex, Brighton BN1 9RH, UK

**Keywords:** approximate Bayesian inference, active inference, PID control, generalised state-space models, sensorimotor loops, information theory, control theory

## Abstract

In the past few decades, probabilistic interpretations of brain functions have become widespread in cognitive science and neuroscience. In particular, the free energy principle and active inference are increasingly popular theories of cognitive functions that claim to offer a unified understanding of life and cognition within a general mathematical framework derived from information and control theory, and statistical mechanics. However, we argue that if the active inference proposal is to be taken as a general process theory for biological systems, it is necessary to understand how it relates to existing control theoretical approaches routinely used to study and explain biological systems. For example, recently, PID (Proportional-Integral-Derivative) control has been shown to be implemented in simple molecular systems and is becoming a popular mechanistic explanation of behaviours such as chemotaxis in bacteria and amoebae, and robust adaptation in biochemical networks. In this work, we will show how PID controllers can fit a more general theory of life and cognition under the principle of (variational) free energy minimisation when using approximate linear generative models of the world. This more general interpretation also provides a new perspective on traditional problems of PID controllers such as parameter tuning as well as the need to balance performances and robustness conditions of a controller. Specifically, we then show how these problems can be understood in terms of the optimisation of the precisions (inverse variances) modulating different prediction errors in the free energy functional.

## 1. Introduction

Probabilistic approaches to the study of living systems and cognition are becoming increasingly popular in the natural sciences. In particular for the brain sciences, the Bayesian brain hypothesis, predictive coding, the free energy principle and active inference have been proposed to explain brain processes including perception, action and higher order cognitive functions [1,2,3,4,5,6,7,8]. According to these theories, brains, and biological systems more generally, should be thought of as Bayesian inference machines, since they appear to estimate the latent states of their sensory input in a process consistent with a Bayesian inference scheme. Given the complexity of exact Bayesian inference, however, approximated schemes are believed to provide a more concrete hypothesis on the underlying mechanisms. One candidate scheme is the free energy principle (FEP), which was introduced in [4] and later elaborated in a series of papers, e.g., [9,10,11], and has its roots in information theory, control theory, thermodynamics and statistical mechanics. While initially the theory emerged in the computational [12] and behavioural/cognitive neurosciences [13,14], over time, further connections with the fields of biological self-organisation, information theory, optimal control, cybernetics and economics among others, have also been suggested [10,15,16,17]. According to the FEP, living systems exist in a limited set of physical states and thus must minimise the entropy of those physical states (see fluctuation theorems for non-equilibrium thermodynamics, e.g., [18]). To achieve this, organisms can minimise the informational entropy of their sensory states, which, under ergodic assumptions, is equivalent to the time average of surprisal (or self-information) [9]. Surprisal quantifies how improbable an outcome is for a system, i.e., a fish out of water is in a surprising state. Biological creatures can thus be seen as minimising the surprisal of their sensations to maintain their existence, e.g., a fish’s observations should be limited to states in water. Since this surprisal itself is not directly accessible by an agent, variational free energy is proposed as an upper bound on this quantity which can be minimised in its stead [4,19]. It has also been suggested that cognitive functions such as perception, action, learning and attention can be accounted for in terms of approximate Bayesian inference schemes such as the FEP. In particular, according to this hypothesis, perception can be described using predictive coding models of the cortex. These models describe perception as a combination of feedforward prediction errors and feedback predictions combined under a generative model to infer the hidden causes and states of sensory data [2]. More recent work has connected these ideas to control theory and cybernetics [15,17,20], extending existing accounts of (optimal) motor control and behaviour [10,13,21,22]. In this view, behaviour is cast as a process of acting on the world to make sensory data better fit existing predictions, with (optimal) motor control cast as a Bayesian inference problem. The most recent attempt to unify predictive coding and optimal control theory usually falls under the name of active inference [10,13].

While in standard accounts of perceptual inference, prediction errors can be suppressed only by updating predictions of the incoming sensations, in active inference, errors can also be minimised by directly acting on the environment to change sensory input to better accord with existing predictions [9,13]. If a generative model encodes information about favourable states for an agent, then this process constitutes a way by which the agent can change its environment to better meet its needs. Thus, under the FEP, these two processes of error suppression allow a system to both infer the properties of, and control, the surrounding environment. Most models implementing the FEP and active inference assume that agents have a deep understanding of their environment and its dynamics in the form of an accurate and detailed generative model. For instance, in [13,23] the generative model of the agent explicitly mirrors the *generative process* of the environment, i.e., the dynamics of the world the agent interacts with. In recent work, it has been argued that this need not be the case [24,25,26,27], especially if we consider simple living systems with limited resources. We intuitively do not expect an ant to model the entire environment where it forages, performing complex simulations of the world in its brain (cf. the concept of Umwelt [28]). When states and parameters in the world change too rapidly, accurate online inference and learning are implausible [29]. This idea is however common in the literature, e.g., [6,13,14,23], where cognition and perception are presented as processes of inference to the best explanation, and agents are primarily thought to build sophisticated models of their worlds with only a secondary role for action and behaviour. A possible alternative introduces action-oriented models entailing a more parsimonious approach where only task-relevant information is encoded [24,25]. On this normative view, agents only model a minimal set of environmental properties, perhaps in the form of sensorimotor contingencies [26], that are necessary to achieve their goals.

The relationship between information/probability theory and control theory has long been recognised, with the first intuitions emerging from work by Shannon [30] and Kalman [31]. A unifying view of these two theoretical frameworks is nowadays proposed for instance in stochastic optimal control [32,33] and extended in active inference [15], with connections to ideas of sensorimotor loops in biological systems [11,13]. These connections emphasise homeostasis, regulation and concepts such as set-point control and negative feedback for the study of different aspects of living systems, with roots in the cybernetics movement [34,35]. It remains, however, unclear how the active inference formulation directly relates to more traditional concepts of control theory. PID control, a popular control strategy working with little prior knowledge of the process to regulate, is commonly applied in engineering [36,37,38] and more recently used in biology and neuroscience modelling [39,40,41,42,43]. In this work, we develop an information theoretic interpretation of PID control, showing how it can be derived in a more general Bayesian (active) inference framework. We will show that approximate models of the world are often enough for regulation, and in particular that simple linear generative models that only approximate the true dynamics of the environment implement PID control as a process of inference. Using this formulation we also propose a new method for the optimisation of the gains of PID controllers based on the same principle of variational free energy minimisation, and implemented as a second order optimisation process. Finally, we will show that our implementation of PID controllers as approximate Bayesian inference lends itself to a general framework for the formalisation of different (conflicting) criteria in the design of a controller, the so-called performance-robustness trade-off [38,44], as a cohesive set of constraints implemented in a free energy functional. In active inference, these criteria will be mapped to precisions, or inverse variances, of observations and dynamics of a state-space model with a straightforward interpretation in terms of uncertainty on different variables of a system.

In Section 2 we will introduce PID control and give a brief overview of the recent literature highlighting the most common design principles used nowadays for PID controllers. The free energy principle will be presented in Section 3, followed by a complete derivation of PID control as a form of active inference. In this section we will also propose that the parameters of a PID controller, its gains, can be optimised following the active inference formulation, which also captures modern design constraints and desiderata of PID controllers.

## 2. PID Control

Proportional-Integral-Derivative (PID) control is one of the most popular types of controllers used in industrial applications, with more than 90% of total controllers implementing PID or PI (no derivative) regulation [38,45]. It is one of the simplest set-point regulators, whereby a desired state (i.e., set-point, reference, target) represents the final goal of the regulation process, e.g., to maintain a room temperature of 23 ${}^{\circ }$C. PID controllers are based on closed-loop strategies with a negative feedback mechanism that tracks the real state of the environment. In the most traditional implementation of negative feedback methods, the difference between the measured state of the variable to regulate (e.g., the real temperature in a room) and the target value (e.g., 23 ${}^{\circ }$C) produces a prediction error whose minimisation drives the controller’s output, e.g., if the temperature is too high, it is decreased and if too low, it is raised. In mathematical terms:

(1) $$\begin{array}{c} e\left(t\right)=y_{r}-y\left(t\right) \end{array}$$

where e(t) is the error, $y_{r}$ is the reference or set-point (e.g., desired temperature) and y(t) is the observed variable (e.g., the actual room temperature).

**Figure 1:**
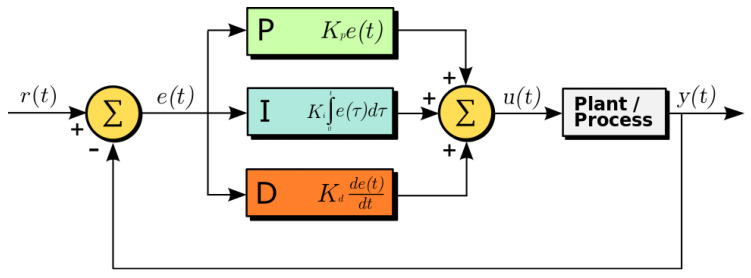
A PID controller [46]. The prediction error e(t) is given by the difference between a reference signal r(t), $y_{r}$ in our formulation, and the output y(t) of a process. The different terms, one proportional to the error (P term), one integrating the error over time (I term) and one differentiating it (D term), drive the control signal u(t).

This mechanism is, however, unstable in very common conditions, in particular when a steady-state offset is added (e.g., a sudden and unpredictable change in external conditions affecting the room temperature which are not under our control), or when fluctuations need to be suppressed (e.g., too many oscillations while regulating the temperature may be undesirable). PID controllers elegantly deal with both of these problems by augmenting the standard negative feedback architecture, here called proportional or P term, with an integral or I and a derivative or D term, see Figure 1. The integral term accumulates the prediction error over time in order to cancel out errors due to unaccounted steady-state input, while minimising the derivative of the prediction error leads to a decrease in the amplitude of fluctuations of the controlled signal. The general form of the control signal u(t) generated by a PID controller is usually described by:

(2) $$\begin{array}{c} u\left(t\right)=k_{p}e\left(t\right)+k_{i}\int_{0}^{t}e\left(\tau \right)d\tau +k_{d}\frac{de(t)}{dt} \end{array}$$

where e(t) is, again, the prediction error and $k_{p},k_{i},k_{d}$ are the so called proportional, integral and derivative gains respectively, a set of parameters used to tune the relative strength of the P, I and D terms of the controller. The popularity of PID controllers is largely due to their simple formulation and implementation. One of the major challenges on the other hand, lies with the tuning of parameters $k_{p},k_{i},k_{d}$, that have to be adapted to deal with different (often conflicting) constraints on the regulation process [36,44].

### 2.1. The Performance-Robustness Trade-Off

The presence of conflicting criteria for the design of PID controller is a well known issue in the control theory literature, often referred to as the performance-robustness trade-off [38,44,47,48,49]. A controller needs to optimise pre-specified performance criteria while, at the same time, preserving some level of robustness in the face of uncertainty and unexpected conditions during the regulation process. In recent attempts to formalise and standardise these general principles [38,44], the performance of a controller has been proposed to be evaluated through:

- load disturbance response, how a controller reacts to changes in external inputs, e.g., a step input,
- set-point response, how a controller responds to different set-points over time,
- measurement noise response, how noise on the observations impacts the regulation process,

while robustness to be assessed on:

- robustness to model uncertainty, how uncertainty on the plant/environment dynamics affects the controller.

The goal of a general methodology for the design and tuning of PID controllers is to bring together these (and possibly more) criteria into a formal and tractable framework that can be used for a large class of problems. One possible example is presented in [48] (see also [50,51] for other attempts). This methodology is based on the maximisation of the integral gain (equivalent to the minimisation of the integral of the error from the set-point, see [36]), subject to constraints derived from a frequency domain analysis related to the Nyquist stability criterion applied to the controlled system [48]. In this work, we propose our formulation also as a general framework for the design and tuning of PID controllers leveraging the straightforward interpretation of the performance-robustness trade-off for PID controllers in terms of uncertainty parameters (i.e., precisions or inverse variances) in the variational free energy.

## 3. The Active Inference Framework

According to the free energy principle, living system must minimise the surprisal, or self-information, of their observations [4,9,10,19], defined as:

(3) $$\begin{array}{c} -\ln p(\psi |m) \end{array}$$

where ψ is a set of sensory inputs conditioned on an agent *m*. Surprisal, in general, can in fact differ from agent to agent, with states that are favourable for a fish (in water), different from those favourable for a bird (out of water) (see [52] for a review on the value of information). According to the FEP, agents that minimise the surprisal of their sensory states over time will also minimise the entropy of their sensations, thus limiting the number of states they can physically occupy [4,19]. This minimisation is, however, intractable in any practical scenario since surprisal can be seen as the negative log-model evidence or negative log-marginal likelihood of observations ψ, with (omitting *m* for simplicity from now on) the marginal likelihood or model evidence expressed as:

(4) $$\begin{array}{c} p\left(\psi \right)=\int_{\vartheta }p\left(\psi ,\vartheta \right)\;d\vartheta . \end{array}$$

This integral is defined over all possible hidden variables, ϑ, of observations ψ. In many cases, the marginalisation is intractable since the latent space of ϑ may be high dimensional or the distribution may have a complex (analytical) form. In statistical mechanics, an approximation under variational formulations transforms this into an optimisation problem. The approximation goes by several names, including variational Bayes and ensemble learning [53,54], and constitutes the mathematical basis of the free energy principle. Using variational Bayes, surprisal can then be decomposed into [54]:

(5) $$\begin{array}{c} -\ln p\left(\psi \right)=F-D_{\mathrm{KL}}\left(q\left(\vartheta \right)\;||\;p\left(\vartheta |\psi \right)\right), \end{array}$$

where

(6) $$\begin{array}{c} D_{\mathrm{KL}}\left(q\left(\vartheta \right)\;||\;p\left(\vartheta |\psi \right)\right)=\int q\left(\vartheta \right)\ln\frac{q(\vartheta )}{p(\vartheta |\psi )}\;d\vartheta , \end{array}$$

is the Kullback-Leibler (KL) divergence [55], or relative entropy [54], an asymmetrical non-negative measure of the difference between two probability distributions. The first one, p(ϑ|ψ), represents the posterior distribution specifying the probability of hidden states, causes and parameters (ϑ) given observations ψ, while the second one q(ϑ), is the variational or recognition density which encodes currents beliefs over hidden variables ϑ. The latter is introduced with the idea of approximating the (also) intractable posterior p(ϑ|ψ) with a simpler distribution, q(ϑ), and then minimising their difference through the KL divergence: when the difference is zero (following Jensen’s inequality, the divergence is always non-negative [54]), q(ϑ) is a perfect description of p(ϑ|ψ). Analogously, from the point of view of an agent, its goal is to explain the hidden states, causes and parameters ϑ of sensations ψ by approximating the posterior p(ϑ|ψ) with a known distribution, q(ϑ). The first term in Equation (5) can be written as

(7) $$\begin{array}{c} F=\int q\left(\vartheta \right)\ln\frac{q(\vartheta )}{p(\vartheta ,\psi )}\;d\vartheta \end{array}$$

and is defined as (variational) free energy [8,12,56,57] for its mathematical analogies with free energy in thermodynamics, or [54] (negative) evidence lower bound in machine learning. Since the KL divergence is always non-negative we arrive at

(8) $$\begin{array}{c} D_{\mathrm{KL}}\left(q\left(\vartheta \right)\;||\;p\left(\vartheta |\psi \right)\right)\geq 0\Rightarrow F\geq -\ln p\left(\psi \right) \end{array}$$

which demonstrates that variational free energy is an upper bound to surprisal, since by minimising *F* we are guaranteed to minimise −lnp(ψ). To evaluate the variational free energy *F*, we must formalise a recognition density q(ϑ) and a generative density p(ϑ,ψ) specific to an agent. Starting from the latter, we define a generative model formulated as a one dimensional *generalised* state-space model [12]:

(9) $$\begin{array}{c} \begin{array}{c} \psi =g(x,v)+z\\ \psi^{\prime }=g_{x}\left(x,v\right)x^{\prime }+g_{v}\left(x,v\right)v^{\prime }+z^{\prime }\\ \psi^{\prime \prime }=g_{x}\left(x,v\right)x^{\prime \prime }+g_{v}\left(x,v\right)v^{\prime \prime }+z^{\prime \prime }\\ \vdots \end{array}\;\;\;\begin{array}{c} \dot{x}=x^{\prime }=f\left(x,v\right)+w\\ {\dot{x}}^{\prime }=x^{\prime \prime }=f_{x}\left(x,v\right)x^{\prime }+f_{v}\left(x,v\right)v^{\prime }+w^{\prime })\\ {\dot{x}}^{\prime \prime }=x^{\prime \prime \prime }=f_{x}\left(x,v\right)x^{\prime \prime }+f_{v}\left(x,v\right)v^{\prime \prime }+w^{\prime \prime })\\ \vdots \end{array} \end{array}$$

where ψ are the observations and ϑ={x,v,θ,γ}, with *x* as the hidden states and *v* as the exogenous inputs, while θ and γ follow a partition in terms of parameters and hyperparameters defined in [12] and are specified later to simplify the notation now. Functions g(·) and f(·) map hidden states/inputs to observations and the dynamics of hidden states/inputs respectively. The prime symbols, e.g., $x^{\prime },x^{\prime \prime },x^{\prime \prime \prime }$ are used to define higher orders of motion of a variable. Generalised coordinates of motion are introduced to represent non-Markovian continuous stochastic processes based on Stratonovich calculus, with strictly non-zero autocorrelation functions [12,58,59]. Ito’s formulation of stochastic processes, on the other hand, is based on Wiener noise, where the autocorrelation can be seen as strictly equal to a delta function [59,60]. In general, the Stratonovich formulation is preferred in physics, where it is assumed that perfect white noise does not exist in the real world [61], while Ito’s calculus is extensively used in mathematics/economics for its definition preserving the Martingale property [62]. It is proposed that models of biological systems should be based on the Stratonovich derivation [12], to accommodate more realistic properties of the physical world (i.e., non-Markovian processes). Using the Stratonovich interpretation, random processes can be described as analytic (i.e., differentiable) and become better approximations of real-world (weakly) coloured noise [60,63,64]. In this formulation, standard state-space models are extended, describing dynamics and observations for higher “orders of motion” encoding, altogether, a trajectory for each variable. The more traditional state-space description is based on Markovian processes (i.e., white noise) and can be seen as a special case of generalised state-space models defined here and in, for instance [8,12]. When coloured noise is introduced, one should either define a high order autoregressive process expressed in terms of white noise [65] or embrace the Stratonovich formulation defining all the necessary equations in a state-space form [12]. The higher “orders of motion” introduced here can be thought of as quantities specifying “velocity” (e.g., ${\left(\psi \right)}^{\prime }$), “acceleration” (e.g., ${\left(\psi \right)}^{\prime \prime }$), etc. for each variable, which is neglected in more standard formulations. For practical purposes, in Equation (9) we also made a local linearity approximation on higher orders of motion suppressing nonlinear terms [8,12]. We introduce then a more compact form:

(10) $$\begin{array}{c} \begin{array}{c} \tilde{\psi }=g\left(\tilde{x},\tilde{v}\right)+\tilde{z} \end{array}\;\;\;\begin{array}{c} {\tilde{x}}^{\prime }=f\left(\tilde{x},\tilde{v}\right)+\tilde{w} \end{array} \end{array}$$

where the tilde sign (e.g., $\tilde{\psi }$) summarises a variable and its higher orders of motion (e.g., $\tilde{\psi }=\left\{\psi ,\psi^{\prime },\psi^{\prime \prime },\cdots \right\}$). The stochastic model in Equation (9) can then be described in terms of a generative density:

(11) $$\begin{array}{c} \begin{array}{c} P\left(\tilde{\psi },\tilde{x},\tilde{v};\theta ,\gamma \right)=P\left(\tilde{\psi }|\tilde{x},\tilde{v};\theta ,\gamma \right)P\left(\tilde{x},\tilde{v};\theta ,\gamma \right) \end{array} \end{array}$$

In this case, we also make the conditional dependence on θ,γ explicit, defining θ as slowly changing parameters coupling hidden states and causes to observations, and hyperparameters γ as encoding properties of random fluctuations/noise $\tilde{w}$ and $\tilde{z}$. P(ψ|x,v;θ,γ) is a likelihood function describing the measurement law in Equation (10), while the prior $P(\tilde{x},\tilde{v};\theta ,\gamma )$ describes the system’s dynamics. Under the Laplace approximation [66,67], the form of the recognition density q(ϑ) is specified in terms of a Gaussian distribution centred around the estimated mode (i.e., the mean for a Gaussian distributions) which can be evaluated using an extension of the EM algorithm [56,57]. Furthermore, (co)variances can be solved analytically in terms of the Hessian of the free energy evaluated at the mode [8,67,68]. The variational free energy in Equation (7) can then be simplified, without constants, to [8]:

(12) $$\begin{array}{c} F\approx -\ln P\left(\tilde{\psi },\tilde{x},\tilde{v};\theta ,\gamma \right){|}_{\tilde{\vartheta }={\tilde{\mu }}_{\vartheta }} \end{array}$$

where the condition $\tilde{\vartheta }={\tilde{\mu }}_{\vartheta }$ represents the fact that the generative density $P(\tilde{\psi },\tilde{x},\tilde{v};\theta ,\gamma )$ will be approximated by a Gaussian distribution centred around the best estimates ${\tilde{\mu }}_{\vartheta }$ of the unknown $\tilde{\vartheta }$, following the Laplace method implemented in a variational context [66]. With Gaussian assumptions on random variables $\tilde{z}$ and $\tilde{w}$ in Equation (10), the likelihood and prior in Equation (11) are also Gaussian, and the variational free energy can be expressed as:

(13) $$\begin{array}{c} \begin{array}{c} F\approx \frac{1}{2}\left[\pi_{\tilde{z}}{\left(\tilde{\psi }-g\left({\tilde{\mu }}_{x},{\tilde{\mu }}_{v}\right)\right)}^{2}+\pi_{\tilde{w}}{\left({\tilde{\mu }}_{x}^{\prime }-f\left({\tilde{\mu }}_{x},{\tilde{\mu }}_{v}\right)\right)}^{2}-\ln\left(\pi_{\tilde{z}}\pi_{\tilde{w}}\right)\right] \end{array} \end{array}$$

where $\tilde{x}$ and $\tilde{v}$ are replaced by their sufficient statistics, means/modes ${\tilde{\mu }}_{x},{\tilde{\mu }}_{v}$, and sensory and dynamics/process precisions $\pi_{\tilde{z}},\pi_{\tilde{w}}$, or inverse variances, of random variables $\tilde{z}$ and $\tilde{w}$. Following [12,56], the optimisation of the (Laplace-encoded) free energy with respect to expected hidden states ${\tilde{\mu }}_{x}$, equivalent to estimation or perception, can be implemented via a gradient descent:

(14) $$\begin{array}{c} {\dot{\tilde{\mu }}}_{x}=D{\tilde{\mu }}_{x}-\frac{\partial F}{\partial {\tilde{\mu }}_{x}} \end{array}$$

while, considering how, from the perspective of agent, only observations ψ are affected by actions *a* (i.e., ψ(a)), control or action can be cast as:

(15) $$\begin{array}{c} \dot{a}=-\frac{\partial F}{\partial a}=-\frac{\partial F}{\partial \tilde{\psi }}\frac{\partial \tilde{\psi }}{\partial a} \end{array}$$

representing a set of coupled differential equations describing a closed sensorimotor loop in terms of a physically plausible minimisation scheme [12]. The first equation includes a term $D{\tilde{\mu }}_{x}$ that represents the “mode of the motion” (also the mean for Gaussian variables) in the minimisation of states in generalised coordinates of motion [8,12,69], with *D* as a differential operator “shifting” the order of motion of ${\tilde{\mu }}_{x}$ such that $D{\tilde{\mu }}_{x}={\tilde{\mu }}_{x}^{\prime }$. More intuitively, since we are now minimising the components of a generalised state representing a trajectory rather than a static state, variables are in a moving frame of reference in the phase-space, and the minimisation is achieved when the temporal dynamics of the gradient descent match the ensemble dynamics of the estimates of hidden states, so for ${\dot{\tilde{\mu }}}_{x}={\tilde{\mu }}_{x}^{\prime }$ rather than for ${\dot{\tilde{\mu }}}_{x}=0$ (which assumes that the mode of the motion is zero, as in standard state-space formulations with Markov assumptions). In the second equation, active inference makes the assumption that agents have innate knowledge of the mapping between actions *a* and observations $\tilde{\psi }$ (i.e., $\partial \tilde{\psi }/\partial a$) as reflex arcs, acquired on an evolutionary time scale, see [13,15] for discussion.

## 4. Results

### 4.1. PID Control as Active Inference

To implement PID control as a process of active inference, we will first describe an agent’s generative model as a generalised linear state-space model of second order (i.e., only two higher orders of motion, anything beyond that is zero-mean Gaussian noise):

$$\begin{array}{c} \begin{array}{c} \psi =x+z\\ \psi^{\prime }=x^{\prime }+z^{\prime }\\ \psi^{\prime \prime }=x^{\prime \prime }+z^{\prime \prime } \end{array}\;\;\;\begin{array}{c} \dot{x}=x^{\prime }=-\alpha \left(x+v\right)+w\\ {\dot{x}}^{\prime }=x^{\prime \prime }=-\alpha \left(x^{\prime }+v^{\prime }\right)+w^{\prime }\\ {\dot{x}}^{\prime \prime }=x^{\prime \prime \prime }=-\alpha \left(x^{\prime \prime }+v^{\prime \prime }\right)+w^{\prime \prime } \end{array} \end{array}$$

where α∈θ is a parameter. As previously suggested, with a Gaussian assumption on $\tilde{z},\tilde{w}$, the likelihood is reduced to:

(16) $$\begin{array}{c} P\left(\tilde{\psi }|\tilde{x},\tilde{v};\theta ,\gamma \right)=P\left(\tilde{\psi }|\tilde{x};\theta ,\gamma \right)=N\left({\tilde{\mu }}_{x},\sigma_{\tilde{z}}^{2}\right) \end{array}$$

where we assume no direct dependence of observations $\tilde{\psi }$ on external inputs $\tilde{v}$, while the prior is described by:

(17) $$\begin{array}{c} P\left(\tilde{x},\tilde{v};\theta ,\gamma \right)=P\left(\tilde{x}|\tilde{v};\theta ,\gamma \right)P\left(\tilde{v};\theta ,\gamma \right) \end{array}$$

with

(18) $$\begin{array}{cc} P(\tilde{x}|\tilde{v};\theta ,\gamma ) & =N(-\alpha \left({\tilde{\mu }}_{x}+{\tilde{\mu }}_{v}\right),\sigma_{\tilde{w}}^{2})\\ P(\tilde{v};\theta ,\gamma ) & =N({\tilde{\eta }}_{x},\sigma_{\tilde{v}}^{2}) \end{array}$$

The Laplace-encoded variational free energy in Equation (13) then becomes:

(19) $$\begin{array}{c} F\approx \frac{1}{2}[\pi_{z}{\left(\psi -\mu_{x}\right)}^{2}+\pi_{z^{\prime }}{\left(\psi^{\prime }-\mu_{x}^{\prime }\right)}^{2}+\pi_{z^{\prime \prime }}{\left(\psi^{\prime \prime }-\mu_{x}^{\prime \prime }\right)}^{2}+\pi_{w}{\left(\mu_{x}^{\prime }+\alpha \left(\mu_{x}-\eta_{x}\right)\right)}^{2}+\\ +\pi_{w^{\prime }}{\left(\mu_{x}^{\prime \prime }+\alpha \left(\mu_{x}^{\prime }-\eta_{x}^{\prime }\right)\right)}^{2}+\pi_{w^{\prime \prime }}{\left(\mu_{x}^{\prime \prime \prime }+\alpha \left(\mu_{x}^{\prime \prime }-\eta_{x}^{\prime \prime }\right)\right)}^{2}-\ln\left(\pi_{z}\pi_{w}\pi_{z^{\prime }}\pi_{w^{\prime }}\pi_{z^{\prime \prime }}\pi_{w^{\prime \prime }}\right)] \end{array}$$

To simplify our formulation, we assume that precisions $\pi_{\tilde{v}}$ tend to infinity (i.e., no uncertainty on the priors for $\tilde{v}$), so that $P(\tilde{v};\theta ,\gamma )$ in Equation (18) becomes a delta function and inputs $\tilde{v}$ reduce to their prior expectations ${\tilde{\eta }}_{x}$, i.e., ${\tilde{\mu }}_{v}={\tilde{\eta }}_{x}$. With this simplification, prior precisions $\pi_{\tilde{v}}$ and respective predictions errors $\left({\tilde{\mu }}_{v}-{\tilde{\eta }}_{x}\right)$ are not included in our formulation (see [56,57] for more general treatments). By applying the gradient descent described in Equations (14) and (15) to our free energy functional, we then get the following update equations for perception (estimation):

(20) $$\begin{array}{cc} {\dot{\mu }}_{x}= & \mu_{x}^{\prime }-\left[-\pi_{z}\left(\psi -\mu_{x}\right)+\pi_{w}\alpha \left(\mu_{x}^{\prime }+\alpha \left(\mu_{x}-\eta_{x}\right)\right)\right]\\ {\dot{\mu }}_{x}^{\prime }= & \mu_{x}^{\prime \prime }-\left[-\pi_{z^{\prime }}\left(\psi^{\prime }-\mu_{x}^{\prime }\right)+\pi_{w^{\prime }}\alpha \left(\mu_{x}^{\prime \prime }+\alpha \left(\mu_{x}^{\prime }-\eta_{x}^{\prime }\right)\right)+\pi_{w}\left(\mu_{x}^{\prime }+\alpha \left(\mu_{x}-\eta_{x}\right)\right)\right]\\ {\dot{\mu }}_{x}^{\prime \prime }= & \mu_{x}^{\prime \prime \prime }-\left[-\pi_{z^{\prime \prime }}\left(\psi^{\prime \prime }-\mu_{x}^{\prime \prime }\right)+\pi_{w^{\prime \prime }}\alpha \left(\mu_{x}^{\prime \prime }+\alpha \left(\mu_{x}^{\prime \prime }-\eta_{x}^{\prime \prime }\right)\right)+\pi_{w^{\prime }}\left(\mu_{x}^{\prime \prime }+\alpha \left(\mu_{x}^{\prime }-\eta_{x}^{\prime }\right)\right)\right] \end{array}$$

and for action (control):

(21) $$\dot{a}=-\left[\pi_{z}\left(\psi -\mu_{x}\right)\frac{\partial \psi }{\partial a}+\pi_{z^{\prime }}\left(\psi^{\prime }-\mu_{x}^{\prime }\right)\frac{\partial \psi^{\prime }}{\partial a}+\pi_{z^{\prime \prime }}\left(\psi^{\prime \prime }-\mu_{x}^{\prime \prime }\right)\frac{\partial \psi^{\prime \prime }}{\partial a}\right].$$

The mapping of these equations to a PID control scheme becomes more clear under a few simplifying assumptions. First, we assume strong priors on the causes of proprioceptive observations ψ. For consistency with previous formulations, e.g., [8,13,15], we will define ψ as proprioceptive observations, where proprioception is the sense of position and movement of different parts of one’s body. For the car model we introduce later, this is equivalent for instance to readings of the velocity of the vehicle. Intuitively, these priors are used to define actions that change the observations to better fit the agent’s desires, i.e., the target of the PID controller. This is implemented in the weighting mechanism of prediction errors by precisions in Equation (19); see also [13,26,70] for similar discussions on the role of precisions for behaviour. In our derivation, weighted prediction errors on system dynamics, $\pi_{\tilde{w}}\left({\tilde{\mu }}_{x}^{\prime }+{\tilde{\mu }}_{x}-{\tilde{\eta }}_{x}\right)$, will be weighted more than weighted errors on observations, $\pi_{\tilde{z}}\left(\tilde{\psi }-{\tilde{\mu }}_{x}\right)$. To achieve this, we decrease sensory precisions $\pi_{\tilde{z}}$ on proprioceptive observations, effectively biasing the gradient descent procedure towards minimising errors on the prior dynamics [70]. Secondly, we set the decay parameter α to a large value (theoretically α→∞, in practice $\alpha =10^{5}$ in our simulations), obtaining a set of differential equations including only terms of order $\alpha^{2}$ for perception:

(22) $$\begin{array}{cc} {\dot{\mu }}_{x}\approx & -\pi_{w}\alpha \left(\alpha (\mu_{x}-\eta_{x})\right)\\ {\dot{\mu }}_{x}^{\prime }\approx & -\pi_{w^{\prime }}\alpha \left(\alpha (\mu_{x}^{\prime }-\eta_{x}^{\prime })\right)\\ {\dot{\mu }}_{x}^{\prime \prime }\approx & -\pi_{w^{\prime \prime }}\alpha \left(\alpha (\mu_{x}^{\prime \prime }-\eta_{x}^{\prime \prime })\right) \end{array}$$

This can be interpreted as an agent encoding beliefs in a world that quickly settles to a desired equilibrium state. This assumption effectively decouples orders of generalised motion, with higher embedding orders not affecting the minimisation of lower ones in Equation (20), since terms from lower orders are modulated by α directly. The remaining terms effectively impose constraints on the generalised motion only close to equilibrium, playing a minor role in the control process away from the target/equilibrium (the more interesting part of regulation). These terms are necessary for the system to settle to a proper steady state when $({\tilde{\mu }}_{x}-{\tilde{\eta }}_{x})\to 0$ and maintain consistency across generalised orders of motion for small fluctuations at steady state, but have virtually no influence at all in conditions far from equilibrium. Following Equation (22), at steady state, expectations on hidden states ${\tilde{\mu }}_{x}$ are mainly driven by priors ${\tilde{\eta }}_{x}$:

(23) $$\begin{array}{c} {\tilde{\mu }}_{x}={\tilde{\eta }}_{x} \end{array}$$

but are still not met by appropriate changes in observations $\tilde{\psi }$ which effectively implement the regulation around the desired target. To minimise free energy in the presence of strong priors, this agent will necessarily have to modify its observations $\tilde{\psi }$ to better match expectations ${\tilde{\mu }}_{x}$, which in turn are shaped by priors (i.e., desires) ${\tilde{\eta }}_{x}$. Effectively, the agent “imposes” its desires on the world, acting to minimise the prediction errors arising at the proprioceptive sensory layers. In essence, an active inference agent implements set-point regulation by behaving to make its sensations accord with its strong priors/desires. After these assumptions, action can be written as:

(24) $$\begin{array}{c} \dot{a}\approx -\left[\pi_{z}\left(\psi -\eta_{x}\right)\frac{\partial \psi }{\partial a}+\pi_{z^{\prime }}\left(\psi^{\prime }-\eta_{x}^{\prime }\right)\frac{\partial \psi^{\prime }}{\partial a}+\pi_{z^{\prime \prime }}\left(\psi^{\prime \prime }-\eta_{x}^{\prime \prime }\right)\frac{\partial \psi^{\prime \prime }}{\partial a}\right] \end{array}$$

where we still need to specify partial derivatives $\partial \tilde{\psi }/\partial a$. As discussed in [13], this step highlights the fundamental differences between the FEP and the more traditional forward/inverse models formulation of control problems in biological systems [71,72]. While these derivatives help in the definition of an inverse model (i.e., finding the correct action for a desired output), unlike more traditional approaches, active inference does not involve a mapping from hidden states $\tilde{x}$ to actions *a*, but is cast in terms of (proprioceptive) sensory data $\tilde{\psi }$ directly. This is thought to simplify the problem: from a mapping between unknown hidden states and actions, to a mapping between known proprioceptive observations $\tilde{\psi }$ and actions *a*. It is claimed that this provides an easier implementation for an inverse model [15], one that is grounded in an extrinsic frame of reference, i.e., the real world ($\tilde{\psi }$), rather than in a intrinsic one in terms of hidden states ($\tilde{x}$) to be inferred first. To achieve PID-like control, we assume that the agent adopts the simplest (i.e., linear) relationship between its actions (controls) and their effects on sensory input across all orders of motion:

(25) $$\begin{array}{c} \frac{\partial \psi }{\partial a}=\frac{\partial \psi^{\prime }}{\partial a}=\frac{\partial \psi^{\prime \prime }}{\partial a}=1\;. \end{array}$$

This reflects a very simple reflex-arc-like mechanism that is triggered every time a proprioceptive prediction is generated: positive actions (linearly) increase the values of the sensed variables $\tilde{\psi }$, while negative actions decrease them. There is, however, an apparent inconsistency here that we need to dissolve: the proprioceptive input ψ and its higher order states $\psi^{\prime },\psi^{\prime \prime }$ are *all* linearly dependent with respect to actions *a* as represented in Equation (25). While an action may not change position, velocity and acceleration of a variable in the same way, a generative model does not need to perfectly describe the system to regulate: these derivatives only encode sensorimotor dependencies that allow for, in this case, sub-optimal control. In the same way, PID controllers are, in most cases, effective but only approximate solutions for control [36,73]. This allows us to understand the encoding of an inverse model from the perspective of an agent (i.e., the controller) rather than assuming a perfect, objective mapping from sensations to actions that reflects exactly how actions affect sensory input [13]. This also points at possible investigations of generative/inverse models in simpler living systems where accurate models are not perhaps needed, and where strategies like PID control are implemented [39,40,41]. By combining Equations (24) and (25), action can then be simplified to:

(26) $$\begin{array}{cc} \dot{a} & \approx \pi_{z}\left(\eta_{x}-\psi \right)+\pi_{z^{\prime }}\left(\eta_{x}^{\prime }-\psi^{\prime }\right)+\pi_{z^{\prime \prime }}\left(\eta_{x}^{\prime \prime }-\psi^{\prime \prime }\right) \end{array}$$

which is consistent with the “velocity form” or algorithm of a PID controller [36]:

(27) $$\begin{array}{c} \dot{u}=k_{i}\left(y_{r}-y\right)+k_{p}\frac{d}{dt}\left(y_{r}-y\right)+k_{d}\frac{d^{2}}{dt^{2}}\left(y_{r}-y\right)\;. \end{array}$$

Velocity forms are used in control problems where, for instance, integration is provided by an external mechanism outside the controller [36,73]. Furthermore, velocity algorithms are the most natural form for the implementation of integral control to avoid windup effects of the integral term, emerging when actuators cannot regulate an indiscriminate accumulation of steady-state error in the integral term due to physical limitations [36,74]. This algorithm is usually described using discrete systems to avoid the definition of the derivative of random variables, often assumed to be white noise in the Ito’s sense (i.e., Markovian processes). In the continuous case, if the variable *y* is a Markov process, its time derivative is in fact not well defined. For this form to exist in continuous systems, *y* must be a smooth (stochastic) process. Effectively, this drops the Markov assumption of white noise and implements the same definition of analytic (i.e., differentiable) noise related to Stratonovich calculus and the generalised coordinates of motion we described earlier. The presence of extra prediction errors beyond the traditional negative feedback (proportional term) can, in this light, be seen as a natural consequence of considering linear non-Markovian processes with simple reflex mechanisms responding to position, velocity and acceleration in the generalised motion phase space (see Equation (25)). To ensure that the active inference implementation approximates the velocity form of PID control we still need to clarify the relationship between the generalised coordinates of motion in Equation (26) and the differential operators $d/dt,d^{2}/dt^{2}$ in Equation (27). As pointed out in previous work, when the variational free energy is minimised, the two of them are equal since the motion of the mode becomes the mode of the motion [8,56]. To simplify our formulation and show PID control more directly, we can consider the case for $\eta_{x}^{\prime }=\eta_{x}^{\prime \prime }=0$, defining the more standard set-point control where a desired or set-trajectory collapses to a single set-point in the state-space and equivalent, in the velocity form, to the case where $y_{r}$ is a constant and $dy_{r}/dt=d^{2}y_{r}/dt^{2}=0$.

**Figure 2:**
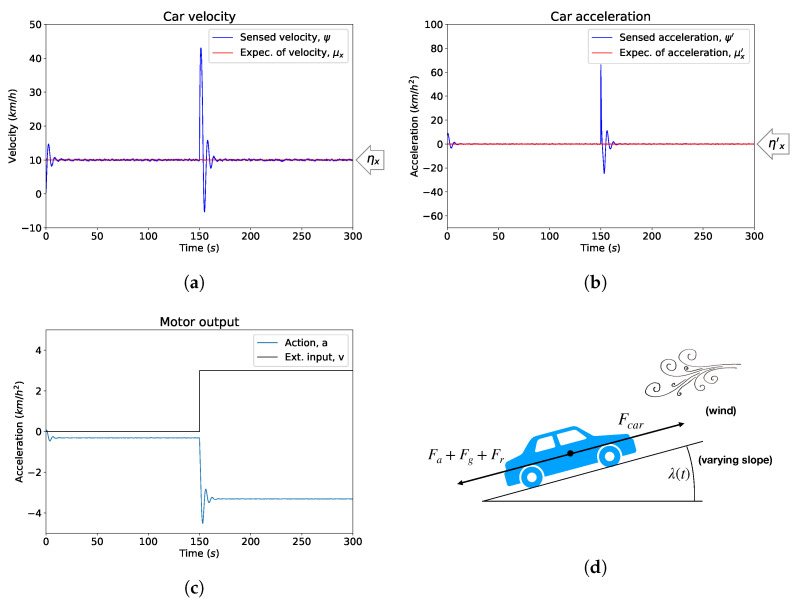
A cruise controller based on PI control under active inference. (**a**) The response of the car velocity over time with a target state, or prior in our formulation, $\eta_{x}=10$ km/h, $\eta_{x^{\prime }}=0$ km/h${}^{2}$; (**b**) The acceleration of the car over time with a specified prior $\eta_{x}^{\prime }=0$ km/h${}^{2}$; (**c**) The external force *v*, introduced at t=150 s, models a sudden change in the environmental conditions, for instance wind or change in slope. Action obtained via the minimisation of variational free energy with respect to *a* and counteracts the effects of *v*. The motor action is never zero since we assume a constant slope, $\lambda =4^{\circ }$ (see Table A1, Appendix A); (**d**) The model car we implemented, where *v* could be thought of as a sudden wind or a changing slope.

To show an implementation of PID control through active inference we use a standard model of cruise control, i.e., a car trying to maintain a certain velocity over time (our code is available at https://github.com/mbaltieri/PIDControlActiveInference.). While only a toy model, the intuitions and results we derive can easily be transferred to the regulation of proteins in bacterial chemotaxis [39] or yeast osmoregulation [75], and more generally to any homeostatic mechanism [34], especially when including limited knowledge of external forces [76]. In this setup, a controller receives the speed of the car as an input and adapts the throttle of the vehicle based on a negative feedback mechanism to achieve the desired, or target, cruising velocity. In real-world scenarios, this mechanism needs to be robust in the presence of external disturbances, essentially represented by changes in the slope of the road, wind blowing, etc., see Figure 2d. For simplicity, we will use the model based on the formulation in [73], see also Appendix A. In this particular instance, we will provide a simple proof of concept, simplifying PID to PI control as in [73], hence implementing only a first order generalised state-space model (see Equation (16)). The controller receives noisy readings $\psi ,\psi^{\prime }$ of the true velocity and acceleration of the car, $x,x^{\prime }$, following the formulation in Equation (16). The controller is provided with a Gaussian prior in generalised coordinates encoding desired velocity and acceleration with means $\eta_{x}=10$ km/h, $\eta_{x}^{\prime }=0$ km/h${}^{2}$. This prior represents a target trajectory for the agent that, as we saw in Equation (26), will be equivalent to integral and proportional terms of a PI controller in velocity form. The recognition dynamics ([69]) are then specified in Equations (20) and (21).

In Figure 2 we show the behaviour of a standard simulation of active inference implementing PI-like control for the controller of the speed of a car. The sensory and process precisions $\pi_{\tilde{z}},\pi_{\tilde{w}}$ are fixed, to show here only the basic disturbance rejection property of PID controllers [36,76]. In Figure 2a, after the car is exposed to some new external condition (e.g., wind) represented in Figure 2c and not encoded in the controller’s generative model, the regulation process brings the velocity of the car back to the desired state after a short transition period. Figure 2b shows how sudden changes in the acceleration of the car are quickly cancelled out in accord with the specified prior $\eta_{x}^{\prime }=0$ km/h${}^{2}$. The action of the car is then shown, as one would expect [76], to counteract the external force *v*, Figure 2c.

### 4.2. Responses to External and Internal Changes

**Figure 3:**
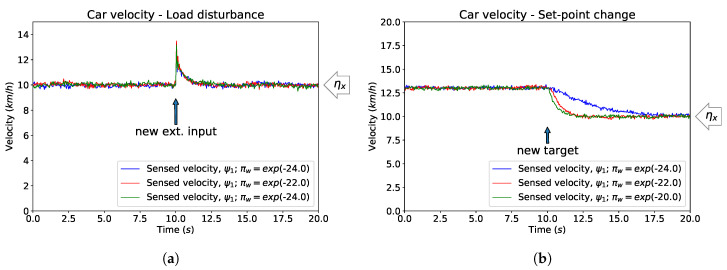
Different responses to load disturbances and set-point changes. The simulations were 300 s long, with an external disturbance/different target velocity introduced at t=150 s. Here we report only a 20 s time window around the change in conditions. (**a**) The same load disturbance (v=3.0 km/h${}^{2}$) is applied with varying expected process precisions $\pi_{\tilde{w}}$ where $\pi_{w}=\left\{\exp\left(-24\right),\exp\left(-22\right),\exp\left(-20\right)\right\}$. Expected sensory log-precisions $\pi_{\tilde{z}}$ are fixed over the duration of the simulations, with $\mu_{\gamma_{z}}=1$; (**b**) A similar example for changes in the target velocity of the car, from $\eta_{x}=13$ km/h to $\eta_{x}=10$ km/h, tested on varying expected process precisions $\pi_{\tilde{w}}$ where $\pi_{w}=\left\{\exp\left(-24\right),\exp\left(-22\right),\exp\left(-20\right)\right\}$.

It is often desirable for a PID regulator to provide different responses to external perturbations (e.g., wind), which should be rather rapid, and to internal updates (e.g., a shift in target velocity) which should be relatively smooth [36,45], see also Section 2.1. It is not, however, trivial to identify and isolate parameters that contribute to these effects [37,77,78], and thus to tune these properties independently. It has been suggested that in order to achieve such decoupling, a controller with two degrees of freedom is necessary [45,77]. Such controller can be thought to contain a feedforward model of the dynamics of the observed/regulated system [73]. In our implementation, this is elegantly achieved by construction, since active inference is based on generative (forward) models. Specifically, we can fix the response to external forces by setting the expected sensory precisions $\pi_{\tilde{z}}$ (i.e., PI gains) but then independently tune the response to changes in the setpoint by altering the expected process precisions $\pi_{\tilde{w}}$ on the priors, see Figure 3a,b.

In the limit for process prediction errors $\pi_{\tilde{w}}\left({\tilde{\mu }}_{x}^{\prime }+\alpha \left({\tilde{\mu }}_{x}-{\tilde{\eta }}_{x}\right)\right)$ much larger than the sensory ones $\pi_{\tilde{z}}\left(\tilde{\psi }-{\tilde{\mu }}_{x}\right)$ and with fixed expected sensory precisions $\pi_{\tilde{z}}$, the response to load disturbances is invariant (Figure 3a). A new target velocity for the car creates different responses with varying $\pi_{w}=\left\{\exp\left(-24\right),\exp\left(-22\right),\exp\left(-20\right)\right\}$ (precisions on higher embedding orders are built, in both cases, using a smoothness (i.e., decay) factor of 1/2, see [12]). Larger $\pi_{\tilde{w}}$ values imply an expected low uncertainty on the dynamics (i.e., changes to the set-point are not encoded and therefore not expected) and are met almost instantaneously with an update of expected hidden states ${\tilde{\mu }}_{x}$, matched by suitable actions *a*. On the other hand, smaller $\pi_{\tilde{w}}$ account for higher variance/uncertainty and thus changes in the target velocity are to be expected, making the transitions to new reference values slower, as seen in Figure 3b.

### 4.3. Optimal Tuning of PID Gains

One of the main goals of modern design principles for PID controllers is to find appropriate tuning rules for the gains on the prediction errors: proportional, integral and derivative terms. However, existing approaches are often limited [37,38,44,48,78]. In general, the proportional term must bring a system to the target state in the first place, the integral of the error should promptly deal with errors generated by steady state inputs not accounted by a model [76], while the derivative term should reduce the fluctuations by controlling changes in the derivative of a variable [73]. In our car example, this could mean, for example, controlling the velocity of the vehicle in spite of changes such as the presence of wind or variations in slope of the road (I term) and avoiding unnecessary changes in accelerations close to the target (D term, even if sometimes not used for cruise control problems [73]). In our treatment of PID controllers as approximate Bayesian inference, the controllers’ gains $k_{i},k_{p},k_{d}$ become equivalent to sensory precisions $\pi_{z},\pi_{z^{\prime }},\pi_{z^{\prime \prime }}$, cf. Equations (26) and (27). Following [12,56,57], we thus propose to optimise these precisions to minimise the path integral of variational free energy (or free action), assuming that parameters and hyperparameters change on a much slower time scale. To do so, we extend our previous formulation and replace fixed sensory precisions $\pi_{z},\pi_{z^{\prime }},\pi_{z^{\prime \prime }}$ with expected sensory precisions $\mu_{\pi_{z}},\mu_{\pi_{z^{\prime }}},\mu_{\pi_{z^{\prime \prime }}}$, derived from a Laplace approximation applied not only to hidden states *x* but extended also to these hyperparameters, now considered as random variables to be estimated, rather than fixed quantities [56,57].

Active inference provides then an analytical criterion for the tuning of PID gains in the temporal domain, where otherwise mostly empirical methods or complex methods in the frequency domain have insofar been proposed [36,38,47,48]. In frameworks used to implement active inference, such as DEM [12,56], parameters and hyperparameters are usually assumed to be conditionally independent of hidden states based on a strict separation of time scales (i.e., a mean-field approximation). This assumption prescribes a minimisation scheme with respect to the path-integral of free energy, or free action, requiring the explicit integration of this functional over time. In our work, however, for the purposes of building an online self-tuning controller, we will treat expected sensory precisions as conditionally dependent but changing on a much slower time-scale with respect to states *x*, using a second order online update scheme based on generalised filtering [57]. The controller gains, $\mu_{\pi_{z}},\mu_{\pi_{z^{\prime }}},\mu_{\pi_{z^{\prime \prime }}}$, will thus be updated specifying instantaneous changes of the curvature of expected precisions with respect to variational free energy rather than first order updates with respect to free action:

(28) $$\begin{array}{c} {\ddot{\mu }}_{\pi_{\tilde{z}}}=-\frac{\partial F}{\partial \mu_{\pi_{\tilde{z}}}} \end{array}$$

Expected precisions $\mu_{\pi_{\tilde{z}}}$ should however be non-negative since variances need to be positive, a fact also consistent with the negative feedback principle behind PID controllers (i.e., negative expected precisions would apply a positive feedback). To include this constraint, following [66] we thus parametrise sensory precisions $\pi_{\tilde{z}}$ (and consequently expected sensory precisions $\mu_{\pi_{\tilde{z}}}$) in the generative model as:

(29) $$\begin{array}{c} \pi_{\tilde{z}}=\exp\left(\gamma_{\tilde{z}}\right) \end{array}$$

creating, effectively, log-normal priors and making them strictly positive thanks to the exponential mapping of hyperparameters γ. The scheme in Equation (28) is then replaced by one in terms of expected sensory log-precisions $\mu_{\gamma_{\tilde{z}}}$:

(30) $$\begin{array}{c} {\ddot{\mu }}_{\gamma_{\tilde{z}}}=-\frac{\partial F}{\partial \mu_{\gamma_{\tilde{z}}}} \end{array}$$

For practical purposes, the second order system presented in Equation (30) is usually reduced to a simpler set of first order differential equations [8]:

(31) $$\begin{array}{cc} {\dot{\mu }}_{\gamma_{\tilde{z}}}= & \mu_{\gamma_{\tilde{z}}}^{\prime }\\ {\dot{\mu }}_{\gamma_{\tilde{z}}}^{\prime }= & -\frac{\partial F}{\partial \mu_{\gamma_{\tilde{z}}}}-\kappa \mu_{\gamma_{\tilde{z}}}^{\prime } \end{array}$$

where $\mu_{\gamma_{\tilde{z}}}^{\prime }$ is a prior on the motion of hyperparameters γ which encodes a “damping” term for the minimisation of free energy *F* (in [57] we can see that this is equivalent to the introduction of a prior $p(\tilde{\gamma })$ on the motion of $\tilde{\gamma }$ to be zero (i.e., zero mean) with precision 2κ). This term enforces hyperparameters to converge to a solution close to the real steady state thanks to a drag term for κ>0 (κ=5 in our simulations). The parametrisation of expected precisions in terms of log-precisions $\gamma_{\tilde{z}}$, in fact, makes the derivative of the free energy with respect to log-precisions strictly positive ($\partial F/\partial \gamma_{\tilde{z}}>0$), not providing a steady-state solution for the gradient descent [57]. This “damping” term stabilises the solution, reducing the inevitable oscillations around the real equilibrium of the system. Given the free energy defined in Equation (19), with $\exp(\mu_{\gamma_{\tilde{z}}})$ replacing $\pi_{\tilde{z}}$, the minimisation of expected sensory log-precisions (or “log- PID gains”) is prescribed by the following equations:

(32) $$\begin{array}{cc} {\dot{\mu }}_{\gamma_{z}}= & \mu_{\gamma_{z}}^{\prime }\\ {\dot{\mu }}_{\gamma_{z}}^{\prime }= & -\frac{\partial F}{\partial \mu_{\gamma_{z}}}-\kappa \mu_{\gamma_{z}}^{\prime }=-\frac{1}{2}\left[\exp\left(\mu_{\gamma_{z}}\right){\left(\psi -\mu_{x}\right)}^{2}-1\right]-\kappa \mu_{\gamma_{z}}^{\prime }\\ {\dot{\mu }}_{\gamma_{z^{\prime }}}= & \mu_{\gamma_{z^{\prime }}}^{\prime }\\ {\dot{\mu }}_{\gamma_{z^{\prime }}}^{\prime }= & -\frac{\partial F}{\partial \mu_{\gamma_{z^{\prime }}}}-\kappa \mu_{\gamma_{z^{\prime }}}^{\prime }=-\frac{1}{2}\left[\exp\left(\mu_{\gamma_{z^{\prime }}}\right){\left(\psi^{\prime }-\mu_{x}^{\prime }\right)}^{2}-1\right]-\kappa \mu_{\gamma_{z^{\prime }}}^{\prime }\\ {\dot{\mu }}_{\gamma_{z^{\prime \prime }}}= & \mu_{\gamma_{z^{\prime \prime }}}^{\prime }\\ {\dot{\mu }}_{\gamma_{z^{\prime \prime }}}^{\prime }= & -\frac{\partial F}{\partial \mu_{\gamma_{z^{\prime \prime }}}}-\kappa \mu_{\gamma_{z^{\prime \prime }}}^{\prime }=-\frac{1}{2}\left[\exp\left(\mu_{\gamma_{z^{\prime \prime }}}\right){\left(\psi^{\prime \prime }-\mu_{x}^{\prime \prime }\right)}^{2}-1\right]-\kappa \mu_{\gamma_{z^{\prime \prime }}}^{\prime } \end{array}$$

This scheme introduces a new mechanism for the tuning of the gains of a PID controller, allowing the controller to adapt to adverse and unexpected conditions in an optimal way, in order to avoid oscillations around the target state.

**Figure 4:**
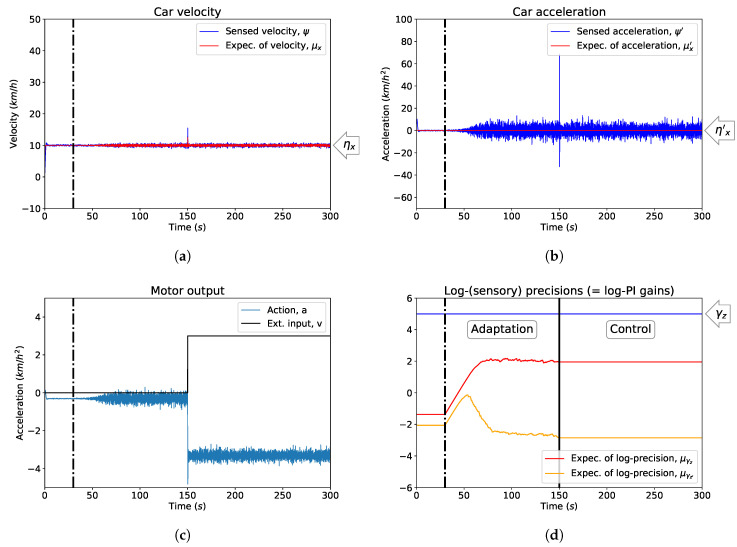
Optimising PID gains as expected sensory log-precisions $\mu_{\gamma_{\tilde{z}}}$. This example shows the control of the car velocity before and after the optimisation of $\mu_{\gamma_{\tilde{z}}}$ (before and after the vertical dash dot black line) is introduced. (**a**) The velocity of the car; (**b**) The acceleration of the car; (**c**) The action of the car, with an external disturbance introduced at t=150 s; (**d**) The optimisation of expected sensory precisions $\mu_{\gamma_{\tilde{z}}}$ and their convergence to an equilibrium state, after which the optimisation is stopped before introducing an external force. The blue line represents the true log-precision of observation noise in the system, $\gamma_{z}=\gamma_{z^{\prime }}=5$.

**Figure 5:**
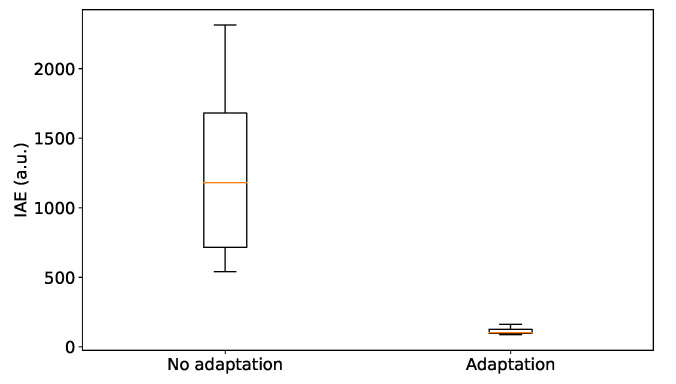
Performance of PID controllers with and without adaptation of the gains based on the minimisation of free energy. The integral absolute error (IAE) is used to measure the effects of the oscillations introduced by a single load disturbance at t=150 s (see text for the exact definition of the IAE).

In Figure 4 the controller for the car velocity is initialised with suboptimal sensory log-precisions $\mu_{\gamma_{\tilde{z}}}$, i.e., log-PI gains. The parameters were initially not updated (Figure 4d) to allow the controller to settle around the desired state, see Figure 4a. The adaptation begins at t=30 s and is stopped at t=150 s, when an external force is introduced, to test the response of the controller after the gains have been optimised. With the adaptation process, the controller becomes more responsive when facing external disturbances (cf. Figure 2), quickly and effectively counteracted by prompt changes in controls, see Figure 4c. As a trade-off, the variances of the velocity and the acceleration are however increased, see Figure 4a,b. The optimisation of the gains through $\mu_{\gamma_{\tilde{z}}}$ without extra constraints (if not the stopping condition we imposed at t=150 s, after the adaptation reaches a steady-state) effectively introduces an extremely responsive controller: cancelling out the effects of unwanted external inputs, such as wind in our cruise control example, but also more sensitive to measurement noise. In Figure 5 we show summary statistics with the results of the adaptation of the gains. Following the examples in Figure 2 and Figure 4, we simulated 20 different cars with expected sensory log-precisions $\mu_{\gamma_{\tilde{z}}}$ sampled uniformly in the interval [−4,−2] and expected process log-precisions $\mu_{\gamma_{\tilde{w}}}$ in the interval [−23,−21]. We initially maintained (i.e., no adaptation) the same hyperparameters and introduced a load disturbance at t=150 s, then repeated the simulations (20 cars) with the same initial conditions allowing for the adaptation of expected sensory log-precisions as log-PI gains after t=30 s, as in Figure 4. Following [79], we measured the performance of the controllers by defining the integral absolute error (IAE):

(33) $$\begin{array}{c} IAE=\int_{t}^{t+\tau }\left|e(t)\right|\;dt \end{array}$$

between two zero-crossings: the last time the velocity was at the target value before a disturbance is introduced, assumed to be t=150 s in our case, and the first time the velocity goes back to the target after a disturbance is introduced (t+τ). To compute t+τ, we took into account the stochasticity of the system and errors due to numerical approximations, considering the case for the real velocity to be within a ±0.5 km/h interval away from the target value. The IAE captures the impact of oscillations on the regulation problem by integrating the error over the temporal interval where the car is pushed away from its target due to some disturbance (for more general discussions on its role and uses see [36]). As we can see in Figure 5, the IAE converges to a single value for all cars (taking into account our approximation of a ±0.5 km/h interval while measuring it) and is clearly lower when the adaptation mechanism for expected sensory log-precisions is introduced, making the controller very responsive to external forces and thus reducing the time away from the target velocity, see Figure 4 for an example.

## 5. Discussion

In this work we developed a minimal account of regulation and control mechanisms based on active inference, a process theory for perception, action and higher order functions expressed via the minimisation of variational free energy [4,8,10,13]. Our implementation constitutes an example of the parsimonious, action-oriented models described in [24,25], connecting them to methods from classic control theory. We focused in particular on Proportional-Integral-Derivative (PID) control, both extensively used in industry [36,37,38,78] and more recently emerging as a model of robust feedback mechanisms in biology, implemented for instance by bacteria [39], amoeba [40] and gene networks [41], and in psychology [42]. PID controllers are ubiquitous in engineering mostly due to the fact that one needs only little knowledge of the process to regulate. In the biological sciences, this mechanism is thought to be easily implemented even at a molecular level [43] and to constitute a possible account for limited knowledge of the external world in simple agents [76].

Following our previous work on minimal generative models [26], we showed that this mechanism corresponds, in active inference terms, to linear generative models for agents that only approximate properties of the world dynamics. Specifically, our model describes linear dynamics for a single hidden or latent state and a linear mapping from the hidden state to an observed variable, representing knowledge of the world that is potentially far removed from the real complexity behind observations and their hidden variables. To implement such a model, we defined a generative model that only approximates the environment of an agent and showed how under a set of assumptions including analytic (i.e., non-Markovian, differentiable) Gaussian noise and linear dynamics, this recapitulates PID control. A crucial component of our formulation is the presence of low sensory precision parameters on proprioceptive prediction errors of our free energy function or equivalently, high expected variance of proprioceptive signals. These low precisions play two roles during the minimisation of free energy: (1) they implement control signals as predictions of proprioceptive input influenced by strong priors (i.e., desires) rather than by observations, see Equation (24) and [13], and (2) they reflect a belief that there are large exogenous fluctuations (low precision = high variance) in the observed proprioceptive input. This last point can be seen as the well known property of the Integral term [73,76] of PID controllers, dealing with unexpected external input (i.e., large exogenous fluctuations). The model represented by derivatives $\partial \tilde{\psi }/\partial a$ encodes then how actions *a* approximately affect observed proprioceptive sensations $\tilde{\psi }$, with an agent implementing a sensorimotor mapping that does not match the real dynamics of actions applied to the environment. The formulation in Equations (20) and (21) can in general be applied to different tasks, in the same way PID control is used in different problems without specific knowledge of the system to regulate.

The generative model we used is expressed in generalised coordinates of motion, a mathematical construct used to build non-Markovian continuous stochastic models based on Stratonovich calculus. Their importance has been expressed before [12,56,57], for the treatment of real world processes best approximated by continuous models and for which Markov assumptions do not really hold (see also [69] for discussion). The definition of a generalised state-space model provides then a series of weighted prediction errors and their higher orders of motion from the start, with PID control emerging as the consequence of an agent trying to impose its desired prior dynamics on the world via the approximate control of its observations on different embedding orders (for I, P and D terms). In this light, the ubiquitous efficacy of PID control may thus reflect the fact that the simplest models of controlled dynamics are first-order approximations to generalised motion. This simplicity is mandated because the minimisation of free energy is equivalent to the maximisation of model evidence, which can be expressed as accuracy minus complexity [10,24]. On this view, PID control emerges via the implementation of constrained (parsimonious, minimum complexity) generative models that are, under some constraints, the most effective (maximum accuracy) for a task.

In the control theory literature, many tuning rules for PID gains have been proposed (e.g., Ziegler-Nichols, IMC, etc., see [36,38] for a review) and used in different applications [36,37,38,48,78], however, most of them produce quite different results, highlighting their inherent fit to only one of many different goals of the control problem. With our active inference formulation, we argue that different criteria can (and should) be expressed within the same set of equations in order to better understand their implications for a system. Modern approaches to the study of PID controllers propose four points as fundamental features to be considered for the design of a controller [44]:

- load disturbance response
- set-point response
- measurement noise response
- robustness to model uncertainty.

In our formulation, these criteria can be interpreted using precision (inverse variance) parameters of different prediction errors in the variational free energy, expressing the uncertainty associated to observations and priors, as reported in Table 1, see also Appendix B for further reference.

**Figure A1:**
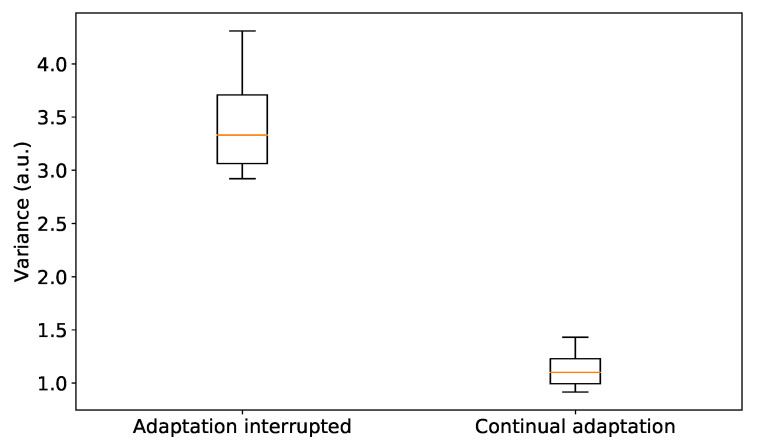
Performance of PID controllers with a sudden increase in measurement noise. Twenty cars simulated in the case where measurement noise is increased at t=150 s during the 300 s simulations. We report aggregate results with the variance from the target value measured over the last 25% (225<t<300 s) of a simulation. We show (1) the case for adaptation of the gains of the PI controller (through expected sensory log-precisions, or log-PI gains, $\mu_{\gamma_{\tilde{z}}}$) interrupted before the measurement noise drastically changes, and (2) the case where the adaptation process persists for the entire duration of the simulations.

After establishing the equivalence between PID control and linear approximations of generalised motion in generative models, we showed that the controllers’ gains, $k_{i},k_{p},k_{d}$, are in our formulation equivalent to expected precisions, $\mu_{\pi_{z}},\mu_{\pi_{z^{\prime }}},\mu_{\pi_{z^{\prime \prime }}}$, for which a minimisation scheme is provided in [12,56,57]. The basic version of this optimisation also produces promising results in the presence of time-varying measurement (white) noise in the simulated car (see Figure A1 in Appendix B). If the adaptation is halted on a system with fixed measurement noise, it can be used to effectively deal with load disturbances, external forces acting against a system reaching his target (see Figure 4), e.g., a change in chemicals concentration for a bacterium.

Future extensions could provide a more principled way of dealing with these two (and possibly other) conflicting cases, an issue that can be solved by introducing suitable hyperpriors (priors on hyperparameters) expressing the confidence of a system regarding changes in measurement noise via the use of precisions on hyperpriors [12]. High confidence (i.e., high precision on hyperpriors) would imply that a system should quickly react to sudden changes, both in measurement noise and other disturbances, since they are unexpected. On the other hand, low confidence (i.e., low precision on hyperpriors) would make a system’s reaction to new conditions slower since such changes are expected. A trade-off between these conditions, with appropriate knowledge of a system or a class of systems introduced in the form of hyperpriors, would then make the process completely automatised, taking advantage of, for instance, empirical Bayes for learning such hyperpriors [10]. By extending our proposition with priors on precisions we can also, in principle, cast more criteria for the controller, expressing different requirements for more complex regulation processes. Given the fact that any optimality criterion can be recast as a prior, following the complete class theorem [80,81], as long as we know how to represent these rules as priors for the controller, we can provide any combination of requirements and tune the parameters in a straightforward fashion.

## 6. Conclusions

PID controllers are robust controllers used as a model of regulation for noisy and non-stationary processes in different engineering fields [38,73]. More recently, they have also been proposed as behavioural models of adaptive learning in humans [42] and as mechanistic explanations of different functions of systems in microbiology [39,40,41]. Their utmost relevance to the natural sciences is becoming clear, with implementations now proposed at the level of simple biomolecular interactions [43,82]. PID controllers are renowned for their simplicity and straightforward interpretation in control theory, however, a general interpretation in probabilistic frameworks (e.g., Bayesian inference) is still missing.

Active inference has been proposed as a general mathematical theory of life and cognition according to the minimisation of variational free energy [10]. On this view, biological agents are seen as homeostatic systems maintaining their existence via the the minimisation of free energy. This process is implemented via the estimation and prediction of latent variables in the world (equivalent to perception) and the control of sensory inputs with behaviours accommodating normative constraints of an agent. Active inference is often described as an extension of optimal control theory with deep connections to Bayesian inference [15]. While methods such as PID control are still widely adopted as models of biological systems, it is unclear how general theories such as active inference connect to practical implementation of homeostatic principles such as PID control. In this work we proposed a way to connect these two perspectives showing how PID controllers can be seen as a special case of active inference. This account is based on the definition of a linear generative model for an agent approximating the dynamics of its environment, potentially very different from the information represented by the model. The model is expressed in generalised coordinates of motion [8,12,69] with prediction errors at different embedding orders for integral, proportional and derivative components emerging naturally as a consequence of an agent assuming non-Markovian dynamics on its sensory input. Through the use of active inference we also proposed the implementation of a mechanism for the optimisation of the gains of a PID controller, i.e., the weights of different prediction errors, now interpreted as precision parameters encoding the uncertainty of different variables from the perspective of an agent.

## Figures and Tables

**Table:** 

.43	.43
(a)	(b)

**Table:** 

.43	.43
(c)	(d)

**Table:** 

.5	.5
(a)	(b)

**Table:** 

.5	.5
(a)	(b)

**Table:** 

.5	.5
(c)	(d)

**Table 1:** Active inference as a general framework for PID controllers.

Criterion	Mapped to	Advantages in Active Inference
Load disturbance response	$\mu_{\pi_{\tilde{z}}}$	Intuitively expressed via the expected inverse variance of the observations (i.e., precision), with low variance implying a fast response and vice versa (see Section 4.2 and Section 4.3)
Set-point change response	$\mu_{\pi_{\tilde{w}}}$	Natural formulation of PID controllers with two degrees of freedom derived from sensory and process precisions and expressed as a Bayesian inference process (see Section 4.2)
Measurement noise response	$\mu_{\pi_{\tilde{z}}}$	Straightforward interpretation of PID gains as (expected) inverse variances of different embedding orders of measurement noise (see Appendix B)
Robustness to model uncertainty	$\mu_{\pi_{\tilde{w}}}$	Direct mapping of model uncertainty to expected variances of the fluctuations, representing unknown dynamics, of the system to control (see Appendix B)

## Appendix A. The Car Model

The equation of motion of the car is:

(A1) $$m\frac{d^{2}s}{dt^{2}}=F-F_{d}$$

where *s* is the position, *F* the force generated by the engine and $F_{d}$ a disturbance force that accounts for a gravitational component $F_{g}$, a rolling friction $F_{r}$ and an aerodynamic drag $F_{a}$, such that $F_{d}=F_{g}+F_{r}+F_{a}$, see again Figure 2d. The forces will be modelled as following:

(A2) $$\begin{array}{c} \begin{array}{cc} F= & r_{g}a\left(t\right)T_{m}\left(1-\beta {\left(\frac{\omega }{\omega_{m}}-1\right)}^{2}\right)\\ F_{g}= & mg\sin\lambda \\ F_{r}= & mg\;C_{r}\mathrm{sgn}\dot{s}\\ F_{a}= & \frac{1}{2}\rho \;C_{d}A{\dot{s}}^{2} \end{array} \end{array}$$

with all the constants and variables reported and explained in Table A1.

**Table A1:** Cruise control problem, constants and variables.

	Description	Value
s(t)	car position	-
$r_{g}$	gear ratio divided by wheel radius	12
a(t)	control	-
$T_{m}$	maximum torque	190 Nm
β	motor constant	0.4
ω	engine speed	$\alpha_{n}v$
$\omega_{m}$	speed that gives maximum torque	420 rad/s
*m*	car mass	100 kg
*g*	gravitational acceleration	9.81 m/s${}^{2}$
λ	slope of the road	$4^{\circ }$
$C_{r}$	coefficient of rolling friction	0.01
ρ	density of the air	1.3 kg/m${}^{3}$
$C_{d}$	aerodynamic drag coefficient	0.32
*A*	frontal area of the car	2.4 m${}^{2}$

## Appendix B. Measurement Noise and Model Uncertainty in Active Inference

Nowadays, it is common to include two more desiderata for the design of PID controllers (see Section 2 and [44]) in order to characterise and tune their response to (1) different types of measurement noise and (2) their robustness to model uncertainty, inherent in simple approximate controllers [38,44]. In our example, these properties map, respectively, to the response of a car given time-varying noise and to the available knowledge of a system, e.g., the working range of a controller or the type of disturbances affecting the car.

In particular, the former describes the behaviour of a PID controller in the presence of noise on the observed variables by modulating the decay of different prediction errors in Equation (26). It is known that this response is (in the limit for t→∞ and with the assumption of a system at equilibrium) inversely proportional to the integral gain [36,38]. In our case, however, we have a more general and trivial relationship where the integral gain $k_{i}$ is, by construction, equivalent to the inverse variance (i.e., precision) of the measurement noise $\pi_{z}$, see Equations (26) and (27). The remaining gains $k_{p},k_{d}$ can then be seen as encoding the uncertainty (i.e., precision) of higher orders of motion when the measurement noise is effectively coloured, otherwise just approximating possible correlations of the observed data over time.

On the other hand, the robustness to model uncertainty is related to expected process log-precisions $\mu_{\gamma_{\tilde{w}}}$ encoding (again by construction) the amplitude of fluctuations due to unknown effects on the dynamics [12]. By modulating the prior dynamics of a system, these hyperparameters assume then a double role, they can either: (1) passively describe (estimate) the dynamics of a system (cf. Kalman filters [83]) or (2) actively impose desired trajectories on observations that can be implemented through actions on the world, as explained in Section 4.1. With these conditions at the extremes, a spectrum of intermediate behaviours is also possible, with $\mu_{\gamma_{\tilde{w}}}$ enacting different sensorimotor couplings by weighting the importance of objective information and desired states/priors of a system.

In the majority of the formulations of control problems, the properties of measurement noise and model uncertainty (especially their (co)variance) are assumed to be constant over time. Often, these parameters also need to be adapted to different systems since their properties are likely to be different. In Section 4.3, we proposed an optimisation method for the gains of a PID controller based on active inference that here we exploit for time changing properties of the noise of a system, and that we show in an example when the measurement noise suddenly increases. In our car example, we could think of a drop in performance of the sensors recording velocity.

We simulated 20 cars for 300 s with adaptation of expected sensory log-precisions (or log-PI gains) $\mu_{\gamma_{\tilde{z}}}$, introduced at t=30 s and stopped at t=150 s. At t=150 s we then decreased the log-precision of measurement noise (n.b. not the expectation on the log-precision) from $\gamma_{z}=5$ to $\gamma_{z}=2$ for the rest of the simulations to simulate the partial failure of a sensor, and stopped the adaptation process. We then simulated 20 cars where adaptation was not halted after the increased measurement noise. To represent the difference, we measured the variance of the real velocity of the cars (without measurement noise to avoid biases), from t=225 s to t=300 s to allow the velocity to settle after the transient due to the sudden change. Agents that kept adapting their gains are shown to be more robust to persistent changes in noise, see Figure A1.

In the case of model uncertainty, given the dual role of $\mu_{\gamma_{\tilde{w}}}$ explained above, i.e., encoding prior dynamics reflecting both real properties of the environment and desired trajectories imposed on the system to regulate, it is harder to show the update of expected precisions without compromising the control of the car. The optimisation of variational free energy is, in fact, not intrinsically biased towards the control of a system, i.e., we externally imposed that as a condition for the agent. While having more flexible priors, an agent could potentially begin to account for uncertainty in the world rather than forcibly changing its observations to reach its target.
